# Current Advances in the Management of Diabetes Mellitus

**DOI:** 10.3390/biomedicines10102436

**Published:** 2022-09-29

**Authors:** Chinyere Aloke, Chinedu Ogbonnia Egwu, Patrick Maduabuchi Aja, Nwogo Ajuka Obasi, Jennifer Chukwu, Blessing Oluebube Akumadu, Patience Nkemjika Ogbu, Ikechukwu Achilonu

**Affiliations:** 1 Protein Structure-Function and Research Unit, School of Molecular and Cell Biology, Faculty of Science, University of the Witwatersrand, Braamfontein, Johannesburg 2050, South Africa; 2 Department of Medical Biochemistry, Alex Ekwueme Federal University Ndufu-Alike, Abakaliki PMB 1010, Nigeria; 3 Department of Biochemistry, Faculty of Biological Sciences, Ebonyi State University, Abakaliki PMB 53, Nigeria; 4 John Hopkins Program on International Education in Gynaecology and Obstetrics, Abuja 900281, Nigeria

**Keywords:** diabetes mellitus, nanotechnology, stem cell, nanomedicine, hyperglycemia

## Abstract

Diabetes mellitus (DM) underscores a rising epidemic orchestrating critical socio-economic burden on countries globally. Different treatment options for the management of DM are evolving rapidly because the usual methods of treatment have not completely tackled the primary causes of the disease and are laden with critical adverse effects. Thus, this narrative review explores different treatment regimens in DM management and the associated challenges. A literature search for published articles on recent advances in DM management was completed with search engines including Web of Science, Pubmed/Medline, Scopus, using keywords such as DM, management of DM, and gene therapy. Our findings indicate that substantial progress has been made in DM management with promising results using different treatment regimens, including nanotechnology, gene therapy, stem cell, medical nutrition therapy, and lifestyle modification. However, a lot of challenges have been encountered using these techniques, including their optimization to ensure optimal glycemic, lipid, and blood pressure modulation to minimize complications, improvement of patients’ compliance to lifestyle and pharmacologic interventions, safety, ethical issues, as well as an effective delivery system among others. In conclusion, lifestyle management alongside pharmacological approaches and the optimization of these techniques is critical for an effective and safe clinical treatment plan.

## 1. Introduction

Diabetes mellitus (DM) is a long-standing, complicated, and non-transmissible endocrine ailment that is growing rapidly and has posed clinical challenges globally, often linked with threats related to complicated metabolic development in patients. It is marked by elevated glucose and lipids in the blood as well as oxidative stress, which culminate in chronic complications involving diverse organs, mainly the kidneys, eyes, nerves, and blood vessels, among others, in the body. As reported by World Health Organization (WHO), DM is an outbreak prone to high malaise and death. Globally, approximately 387 million persons are affected by this disorder and it is estimated to be more than 640 million by 2040 [1].

According to a report in 2017 by International Diabetes Federation (IDF), 425 million persons suffer from diabetes mellitus out of which more than 90 percent are adults and 352 million had impaired glucose tolerance (IGT) [2]. In individuals suffering from type II diabetes mellitus (T2DM), hyperglycemia is not the only characteristic; it also involves multiple complications such as kidney failure, blindness, heart attack, stroke, and amputations of the lower limb [3]. Mounting evidence obtained from epidemiological studies has shown that T2DM is an ailment with numerous causes associated with both polygenic and various environmental factors [4]. T2DM is thus too complicated to cure due to genetic polymorphism and other numerous risk factors.

Despite the fact that most cases are a result of obesity-linked T2DM, the annual prevalence of T1DM is on the rise [5]. It has been reported that about 10 percent of people suffering from diabetes have T1DM. However, the two forms are linked with a prolonged risk of circulatory system complexities [6] and the threat of lowered blood glucose. Ample proof suggests that normoglycemia accomplishment will mitigate the risk of complications linked with DM [7]. However, hypoglycemia occurrences limit the attainment of near normoglycemia in subjects with T1DM. Diabetic individuals who are not aware of their hypoglycemic status are vulnerable to T1DM which then limits them from the attainment of the needed glycemic control. Globally, DM health centers have several individuals with T1DM who have recurrent low blood glucose and the idea of hypoglycemic unconsciousness poses critical clinical challenges. Providentially, many favorable and interesting gain ground exist in the perspective for subjects with the problem of DM, including gene therapy, as reported by Bosch and colleagues [8].

Currently, the main therapeutic regimens for T2DM are injection of insulin-like agents and oral administration of hypoglycaemic agents. However, these agents play crucial functions in T2DM treatment but are laden with side effects [9,10]. Insulin has taken the centerpiece for the management of unrestrained insulin-deficient DM since its invention [11]. Admittedly, due to the severe lack of beta cells, the injection of exogenous insulin is vital for survival. Notwithstanding the advances made in comprehending the etiology, effects, and continuance of DM, including the progress made in insulin development and its analogues, ensuring tight glycaemic modulation without negative side effects such as low blood glucose and gain in weight still poses significant problems [7,12,13]. Hence, this further accentuates the importance of alternative techniques or adjuncts to insulin [14].

Consequently, this narrative review exploits different alternative therapeutic regimen for the management of two forms of DM, including nanotechnology, stem cell technology, gene therapy, medical nutrition therapy, lifestyle modification and the challenges associated with these techniques.

## 2. Methods

To identify published works on recent advances in the management of DM, the literature search for this narrative review was carried out using different search engines including Scopus, Google Scholar, Pubmed/Medline and Web of Science databases. Keywords and subject headings employed include diabetes mellitus, hyperglyceamia, management of DM, T2DM, nanotechnology in diabetes, gene therapy in DM management and current treatment, etc. The titles and abstracts of the results after the search were painstakingly screened to select eligible articles for full-text reading. Articles that were found to be eligible were retrieved and full-text screening was performed independently by three of the authors to select studies for inclusion in the final analysis. Original research and review articles published between 1993 and 2022 (in English) were included. Unpublished articles and thesis were excluded. All authors confirmed the validity of the selected papers.

## 3. Risk Factors of Diabetes

There are several risk factors associated with diabetes. These risk factors contribute significantly to the progression of diabetes. They include but not limited to age; weight; family history of diabetes; smoking and race/ethnicity [15,16] (Asiimwe et al., 2020; Noh et al., 2018). While T1DM is mostly found in the young, T2DM is an adult-related condition. The risk of T2DM increases with age which is due to the deficiency of insulin secretion which develops with age, and growing insulin resistance caused by a change in body composition [17]. Increase in body weight which leads to obesity is closely associated with diabetes in a condition termed diabesity. This is because increase in body weight leads to increased insulin resistance [18].

According to the FDA, smokers are 30 to 40% more likely to come down with T2DM than nonsmokers. Smoking can also increase insulin resistance which makes the patients require more insulin for the control of their sugar level [19]. Diabetes is hereditary. Those with the family history are advised to adhere to lifestyles that reduce the risk of developing diabetes.

## 4. Management of Diabetes

There are several modern approaches involved in the management of diabetes. However, early diagnosis is central to achieving any targets set in DM management [20]. Each patient is treated with the aim of achieving a particular outcome. These outcomes are set out from the first day of clinic visit to ensure an individualized approach in the management of diabetes.

### 4.1. Internet Intervention for Lifestyle Modification in Diabetes

Lifestyle modification is an integral part of diabetes management. It is recommended for both patients in pre-diabetic and diabetic conditions, respectively. Reduced sedentary lifestyle, increased physical activities, and healthy diets are among the recommended lifestyle modifications. The right exercise may depend on the state of the patient. The exercise helps to bring down the plasma glucose level. For a healthy diet, it is recommended that diabetic subjects take a lot of vegetables, fruits, and whole grains; choose nonfat dairy and lean meats; and limit foods that are high in sugar and fat. Other lifestyle changes include stopping smoking and reduction in alcohol intake [21,22]. The lifestyle changes are usually individualized.

Even though the above strategies help in the effective management of diabetes, communicating or constantly reminding the subjects to complete them could be challenging. Web or internet-based program have been deployed to improve adherence to the lifestyle changes. These web-based strategies provide a viable option for facilitating diabetes self-management [23].

### 4.2. Nanotechnology and Diabetes

Nanotechnology involves the use of nanoparticles (<100 nm). These nanoparticles are developed through the manipulation of individual atoms or molecules in a substance. The application of nanotechnology in medicine is termed nanomedicine. Nanomedicine involves the combination of the knowledge of nanotechnology in the application of drugs or diagnostic molecules which generally improves their ability to target specific cells or tissues. Nanotechnology in diabetes research has played several roles in improving the outcome of diabetic management in diabetics through the deployment of novel nanotechnology-based glucose measurement and insulin delivery techniques [24,25]. Nanotechnology employs non-invasive approaches for insulin delivery and the development of a more efficacious vaccine including cell-based and gene-based therapies for T1DM [24]. The importance of nanotechnology in diabetes includes, but is not limited to, inventive diabetes diagnosis, detection of immune cell activity and beta-cell mass, monitoring of glucose level, and non-invasive insulin delivery, etc.

Early and accurate diagnosis of a disease may be as important as the treatment of the disease itself. Prompt diagnosis may prevent dysglycaemia and reduce the time to onset of diabetes [26]. Conventional approaches have been utilized in the different diagnostic needs in diabetes, such as detection of immune destruction that precedes T1DM and/or measurement of plasma glucose levels. However, the shortcomings of the conventional approaches which include, but are not limited to, non-early detection of the disease progression necessitate the need for a novel technology that can improve the diagnostic outcome.

The mass of the beta cell is an indication of the functionality of the beta-cell in secreting insulin. The progressive loss of the beta cells precipitates T1DM [27]. Prompt detection of the stage of beta cell loss through nanotechnology can allow for the immediate application of clinical interventions for its arrest. Magnetic nanoparticles (MNPs), for instance, have distinctive physical properties qualifying them as outstanding contrast media for magnetic resonance imaging (MRI). This can enable the early detection of the stages of beta-cell loss.

Glycaemic fluctuation should be avoided during diabetic management. Individuals have treatment goals set by their physicians. Regular or daily glucose monitoring is performed to ascertain the control achieved by the treatment and the diabetes progression [28]. However, this comes with some challenges including poor compliance as a result of the regular pricking of the patients and inability to monitor glucose levels at certain times of the day (e.g., sleeping and driving times). The overall impact is irregular monitoring of the glucose level which can lead to dangerous fluctuations that may worsen diabetic complications. To circumvent this challenge, continuous glucose monitoring (CGM) systems are essential. The implantation of biosensors (e.g., amperometric sensors) subcutaneously had been used to achieve CGM for 10 days; however, this has its drawbacks including instabilities and the need for a weekly change of the implantation [29,30].

Nanomedicine can overcome the aforementioned obstacles in CGM. The glucose-sensing device has three key components: a detector, a transducer, and a reporter. The detector measures the glucose level while the transducer converts the measurement into an output signal. The reporter finally processes the signal into an interpretable form. For an effective measure of the glucose level, the glucose sensors are usually made of nanoparticles in nanotechnology which are made from mainly three molecules: glucose oxidase, glucose-binding proteins, and glucose-binding small molecules [24,31]. The coupling of these nanoparticles as transducers enables the accurate detection of glucose in a patient-friendly and rapid manner [31].

Insulin shots are the mainstay in the management of T1DM and T2DM. The conventional approach of insulin delivery involves the use of needle injections. Even though some needles have been significantly improved to be painless during delivery, the thought of needles alone could be discouraging [32]. This significantly affects the compliance of patients to insulin use. Moreover, the lingering time between the time of glucose measurement and the insulin dosing in addition to the hindrance in the absorption of insulin ensuing the conventional subcutaneous injection, do not allow for a close plasma glucose control which leads to fluctuations and times of hyperglycemia [24]. An approach that is non-invasive will be well accepted by both patients and medical practitioners to improve compliance and the overall outcome of treatment.

To overcome the recent delivery challenges faced by the conventional approaches, microcomputer closed-loop or nano pumps are being developed to ensure the timely delivery of insulin while ensuring continuous glucose monitoring. In other words, this system is built to link insulin delivery to plasma glucose concentration. This will prevent the risk of plasma glucose fluctuations [26,33]. Other less invasive means of insulin delivery that involve the use of nanoparticles are also being explored to facilitate insulin delivery orally, transdermally, and/or via inhalation [26].

### 4.3. Medical Nutrition Therapy in Diabetes

Medical nutrition therapy (MNT) is a “nutrition-based treatment provided by a registered dietitian nutritionist.” It comprises nutrition diagnosis and therapeutic and professional counseling services that aid in the management of DM. MNT is a critical aspect of diabetes education and management. Recommendations on MNT by international collaborative groups for diabetes management have attempted to reform and provide courses for adverse nutritional transition [34,35]. For instance, MNT has been employed for the treatment of GDM because carbohydrate (CHO) is the main causative agent as a result of its impact on glycaemia. According to the Institute of Medicine, pregnant women require a minimum of 175 g CHO per day, and low-CHO diets already in use traditionally for the treatment of GDM have proven to be safe [36]. Moreover, MNT has been reported to be critical in the management of other types of DM and as such has significantly impacted patients, especially women and newborns [37]. Primarily, MNT ensures the maintenance of euglycemia via adequacy in weight gain in pregnancy and growth of fetus while avoiding ketogenesis and metabolic acidosis. Nonetheless, MNT is yet to establish the optimal diet in terms of energy content and macronutrient distribution, quality, and amount, among others, in DM [37]. Reports have shown that the nutritional requirements for GDM patients are the same for all pregnancy cases when their carbohydrate intake is taken into special cognizance. Currently, a low-glycemic index diet has been reported to be more favorable in the management of GDM than the traditional intervention of carbohydrates restriction even though the evidence is still restrained [37]. Caloric restrictions are very vital in the management of overweight or obesity.

Reports have charged MNT with the design of signature diet strategies that will be suitable medically as well as patient focused. By this, it is hoped that practicing diabetologists and registered dieticians (RDs) will partner to furnish nutritional guidelines based on evidence for use by MNT for the prevention and management of DM and related comorbidities [38]. Indications show that MNT may be a potent, easily available, and cheap therapeutic technique and could be an essential tool for DM prevention and management [35].

### 4.4. Gene Therapy and Diabetes Mellitus

Gene therapy is a technique that involves remedying the symptoms of an ailment orchestrated by a defective gene via the incorporation of the exogenous normal gene. Its advantage is that a single treatment can be used to cure any type of disease and currently, gene therapy is opening up novel treatment options in different branches of medicine [39]. At present, gene manipulation is not limited to the addition of a gene but also gene modulation and editing [40,41]. Gene therapy can also be explained as a method of introduction of a gene or gene manipulation within a cell as a curative regimen in the treatment of disease [42]. The objective of this approach is to remedy abnormal genes that have been implicated as the causative agent in any ailment and to successfully halt the beginning of the ailment or prevent its continuation. The gene therapy approach involves three major intervention methods: (i) delivery of a new gene into the body, (ii) substitution of the abnormal gene with a working gene, and (iii) disabling the malfunction genes responsible for the ailment [42,43]. Gene therapy can be further classified into somatic gene therapy or germline gene therapy. While the primary target in somatic gene therapy is the somatic cells often referred to as the diseased cells, the reproductive cells are the targets in germline gene therapy. Germline therapy halts the development of the disease in subsequent generations [43]. The application of gene therapies as trends in evolving therapeutics is due to its potential for the treatment of diverse ailments including DM, autoimmune disorders, heart diseases, and cancers among others that are difficult to manage using conventional therapies [44].

T1DM is an autoimmune ailment marked by T-cell-orchestrated self-damage of the islet beta cells responsible for the secretion of insulin. Its management is problematic and complex, particularly using conventional drugs. Thus, gene therapy is partly an emerging promising therapeutic alternative in its treatment [45,46]. The etiology of T1DM is multifactorial involving both environmental and genetic factors akin to any other autoimmune disease. In the recent past, researchers have favourably pointed out many genes accountable for the evolution of T1DM [47]. Thus, alteration or grappling with these genes employing gene therapy techniques will probably foster better comprehensible management of the ailment or even cure T1DM.

Even though gene therapy for DM majorly centres on T1DM, many genes have been evaluated as a probable treatment for T2DM as the ailment has a compelling genetic susceptibility [48]. About 75 independent genetic loci have been identified for T2DM via genetic linked studies and different novel therapeutic targets have also been determined [46]. Genetic loci might have a huge impact on drug response in contrast to the incidence and development of diseases whose effects are limited [49]. Many genetic loci exist with prospects for T2DM gene therapy. For instance, nucleotide-binding oligomerization domain-like receptor protein 3 (NLRP3) is a good example. NLRP3 inhibition mitigates inflammation, guard against apoptosis of pancreatic b-cells including the prevention of development of T2DM in mice [50]. Hypothetically, all genes associated with the beginning, evolution, and deterioration of T2DM are probable targets. In Table 1 [51], the genes that modulate the homeostasis of glucose, ameliorate insulin synthesis or/and responsiveness, and improve diabetic mellitus-induced complications are abridged for simplicity.

### 4.5. Stem Cell Therapy in Diabetes

The conventional approaches in the management of DM do not resolve the causes of the ailment and are laden with adverse effects. Hence, there is a quest for a desirable different therapeutic regimen. The cellular-based therapeutic technique currently in use in DM management is based on the pancreas or islet-cell transplantation to revive the beta cells for insulin secretion. This approach is restricted due to a lack of donor organs. These problems lead to the exploration of the possibility of constructing beta cells using stem cells. The peculiar rebuilding potential of stem cells might be an important tool that could be used in the management of DM. Development of replenishable islets source using stem cells might avert the recent supply/demand problems in the transplantation of islet and furnish DM subjects with a prolonged source of beta cells for insulin secretion. Hence, in the management of DM, stem cell investigation has become a promising approach [52].

The stem cell DM therapy is aimed at the replacement of malfunctioning or damaged pancreatic cells by employing pluripotent or multipotent stem cells. This technique has exploited the ability of various kinds of stem cells including induced pluripotent stem cells (iPSCs), embryonic stem cells (ESCs), and adult stem cells using diverse methods to produce surrogate beta cells or to bring back the physiologic role of the beta cell [53].

Advancement in technology has facilitated the development of stem cells using different kinds of tissue sources such as adipose tissue, skin, bone marrow, umbilical cord blood, periosteum, and dental pulp. In searching for promising stem cells, the first organ of choice is usually the pancreas. Studies with animal models have indicated that a small number of pancreatic tissue when made available could bring back the optimum pancreatic beta-cell mass [54]. This is sequel to the differentiated beta cells from the pancreatic duct undergoing replication and dedifferentiation culminating in the formation of pluripotent cells which in turn synthesize more beta cells. Additional study suggested that these ductal cells populations could be produced in vitro and directed to produce insulin synthesizing clusters [55,56].

Moreover, the haemopoietic adult stem cells such as HSCs and mesenchymal stem cells (MSCs) have the potential to transdifferentiate into so many cell lineages such as the brain, liver, and lung as well as gastrointestinal tract cells [57,58,59]. A different group of researchers experimented on the multipotent differentiation of haemapoietic progenitors to replenish the beta cell number in T1DM. It was reported that the bone marrow of mouse was differentiated ex vivo into functional beta cells [60]. Relatedly, studies using the mice model indicated that cells of the bone marrow could be amenable to the pancreas as a target and that elevated blood glucose could be normalized [61]. An experiment with autologous HSCs demonstrated an improvement in T1DM and T2DM [62,63]. These studies furnish potential outcomes for the usage of autologous HSCs in the management of DM.

### 4.6. Latest Inventions in Diabetes Management

In addition to the aforementioned innovations in the management of diabetes, several drugs are still at different stages of clinical trial for eventual use. Others are ready and have been recently introduced into the market.

#### 4.6.1. Drugs Recently Introduced

Tirzepatide: The drug was recently approved by the FDA under the trade name mounjaro for the treatment of T2DM [64]. Tirzepatide is an injectable given under the skin once in a week which targets the receptors of hormones which play central role in the metabolism of glucose. These hormones are glucagon-like peptide-1 (GLP-1) and glucose-dependent insulinotropic polypeptide (GIP). While the GLP-1 reduces blood glucose by several mechanisms, including stimulating insulin secretion and suppressing glucagon release during hyperglycemia, GIP stimulates insulin release during hyperglycemia, but it also stimulates glucagon release during hypoglycemia.

Tirzepatide acts as agonist to their receptors [65], hence elongating their functions which results in blood glucose control. The efficacy of tirzepatide was established against a placebo, a GLP-1 receptor agonist (semaglutide) and two long-acting insulin analogs either as monotherapy or in combination with other antidiabetic agents [64]. In comparison to the placebo, it lowered the HbA1c by 11.6% and 1.5% as monotherapy and combination therapy, respectively. In comparison to other antidiabetic drugs, at the highest dose of 15 mg, it lowered the HbA1c 0.5% more than semaglutide, 0.9% more than insulin degludec and 1.0% more than insulin glargine [64]. Because of the efficacy therein and the once in a week dosing, tirzepatide provides a desirable paradigm shift in the management of T2DM.

#### 4.6.2. Drugs in the Pipeline

Several drug candidates are at different phases of development for the management of DM. These are listed below.

LY3502970: LY3502970 is a partial agonist, biased toward G-protein activation over β-arrestin recruitment at the (GLP-1 receptor (GLP-1R). The molecule is highly potent and selective against other class B G-protein-coupled receptors (GPCRs) with a pharmacokinetic profile favorable for oral administration [66]. It is a product that is currently being developed by Eli lilly.

SCO-094: SCO-094 is a drug candidate identified by SCOHIA company which has a dual target of the receptors of GIP and GLP-1 [67]

Ladarixin (LDX): Ladarixin is an inhibitor of the interleukin-8 receptors CXCR1 and CXCR2, in new-onset T1DM [68]. It is a drug candidate developed by Dompe Farmaceutici. Short term LDX treatment of newly diagnosed patients with T1DM had no appreciable effect on preserving residual beta cell function [68].

## 5. Discussion of Major Findings

DM is a complex, progressive, and multifactorial metabolic disorder needing more complex treatments over time. Globally, researchers have worked assiduously in the discovery and development of novel drugs for the treatment of diabetes. There is significant progress in research into the cause and management of T1DM [69]. Mounting evidence indicates that modern insulin therapy in combination with glucose self-monitoring including blood pressure and lipid monitoring has profoundly improved the long-term prognosis of T1DM [70]. The literature indicates that regular exercise and improved diet may enhance the quality of life for diabetic subjects but in the absence of adequate exercise and diet, medications may help diabetic persons regulate their blood glucose level. Moreover, implantation of insulin producing cells could furnish the basal glucose level essential for maintaining glucose homeostasis in vivo and thus hinder long-term injury from occurring in different tissues regardless of hormone administration [71].

The attainment of the full potential of gene therapy technique could be obtained via the design of gene delivery vectors that are safe, efficient, and specific and/ or the development of a technique for engineering of cell, in which the stem cell seems to be of great importance. Thus, the establishment of a reliable, sensitive, and acutely monitored feedback system is needed for the generation of a safe and efficient vector to facilitate diabetes gene therapy for clinical trial. Probably, the curtailment of islet transplantation rejection is the first clinical technique to DM gene therapy approach. On the other hand, insulin gene therapy is carried out in concert with conventional insulin treatment culminating in tight glycemic regulation in the absence of fasting hypoglycemia in T1DM subjects, as reported in T1DM rats [72].

Physical activity and nutrition therapy could help individuals with DM achieve metabolic goals. Employing diverse lifestyle approaches might help. Regulation of metabolic parameters such blood pressure, glucose, glycated hemoglobin, lipids, and body weight including the assessment of life quality are critical in determining the level of treatment goals by lifestyle changes [73]. However, different countries have focused on DM management and its complications on the normalization of glycemic control as assessed by hemoglobin A1 or fasting blood glucose which only addresses the need of subjects who were already diabetic. Thus, it is imperative to design programs for the early detection of altered glucose metabolism and to carry out robust approaches for the normalization of this changed state. Furthermore, through robust prevention strategies, better diagnostic tests, early risk detection, and management of the risks will help mitigate the incidence of DM and reduce or prevent events associated with end-organ failure [73].

Besides glycemic control, multifactorial interventions using different treatment regimen, including nanotechnology, gene therapy, stem cell, medical nutrition therapy, and lifestyle modification have yielded significant results in ameliorating the impact of DM but not without some challenges. Regardless of the promising nature of nanotechnology and its projected ability to turn around the fortunes in diabetes management, it is still faced with some challenges. One of the major limitations is the cost. Most of the gadgets required for CGM, and insulin delivery are very expensive. This limits their use to the rich class even when diabetes cuts across different economic classes. More so, there is an increased risk of infection via the implantation of sensors and cannulas which increases inflammation and could be frightening sometimes [24].

Notwithstanding the merits linked with the gene therapy approach, there could equally be problems. For example, genes introduced employing a viral vector might provoke an immune response and aggravate the disease condition [74]. Additionally, gene therapy studies are still mostly carried out using animal models and their safety is yet to be validated in humans [46].

Currently, it is established that gene delivery technology is the primary hurdle for successful gene therapy. The prime factors for an effective gene delivery technique include efficiency, stability, specificity, safety, and convenience. Thus, the greatest obstacle in gene therapy is the method of delivery of the corrective gene to the target site safely and efficiently. There is, therefore, a requirement of desirable gene delivery technology or vector to furnish the therapeutic potential where required. The two main vectors currently employed are viral and non-viral vectors. The merit of the non-viral vector is that it has low immunity, a low financial burden, and its preparation is convenient but the major obstacles for its extensive use emanate from the inefficiency of delivery method and expression of gene transiently [75]. Contrastingly, reports show that viral vectors are more efficient in gene delivery as several of them use a distinct mechanism for DNA delivery to the cells. Viral vectors are arranged as viral particles having precisely the important modulated sequences of the virus and from which all the genes of the virus have been excised. These viruses, when prepared very well, are defective that after target cell infection, there is no probable replication or infection theoretically [76]. Viral DNA is integrated with the genome of the host cell, thereby bestowing the capability for sturdy therapeutic gene expression.

Despite the fact that viral vectors are more efficient in comparison to non-viral vectors as gene delivery systems, there are still challenges associated with them, including inflammation, cytotoxicity, and immunogenicity which are needed to be looked into during the construction of viral vector system [46].

Notwithstanding the huge and novel impacts recorded in the applicable areas of stem cell biology in the management of DM, it is still in its primitive stage. A lot of hurdles still hinder the progression of stem cell research technologically and ethically, including:

The use of ESCs is confronted with the formation of teratomas and the danger of malignancy [77], thus raising safety concerns. This makes it imperative for a thorough investigation and screening of the probable adverse effects prior to its deployment in clinical trials and human treatment.

The primary hurdle associated with transplantation is autoimmune rejection. This makes it necessary for a stable and appropriate regimen for immunosuppression. There is a need for the stabilization of current transplantation protocols with the standard testing module. The transplantation of stem cells needs a few experimental works to appraise the problems linked with the stability, durability, and the survival of the transplanted cell with appropriate vascular and neural support in the new microenvironment.

The challenges of scale-up problems arise after the optimization of the appropriate developmental procedures. The number of cells must be enough to cope with the requisite request for future research including clinical investigations. Hence, an efficient method is required for the maximization of the yield via an adjustment in the culture requirements. The stem cells’ scale-up ability is needed for future exploration for the provision of surplus transplanted cellular reserves in order to strike equilibrium between demand and usage.

As a result of where it is obtained from, the ESCs are the potential targets for the ethicists. Normally, ESCs are obtained from embryos not fertilized or used during ex vivo fertilization in hospitals. Informed consent is usually required in the procurement of these ESCs from the donor prior to the usage in clinical research. Sadly, though, in the majority of instances, there is the destruction of the embryo during the process of obtaining the cells from the embryo, and this questions the source of life and the ethical license to terminate the fetus. Adult stem cells are preferable to embryonic ones as the controversy about their usage is limited. The current advancement in technology in induced pluripotent stem cell research is to allow the use of ones’ stem cells for diverse uses [78]. The adult cells are reprogrammed in such cases to pluripotent conditions and thereafter transformed into working beta cells. This approach might eventually resolve the impasse linked with ESCs and contribute to further safety issues likely to be tackled later in the future.

## 6. Conclusions

DM has become a public clinical challenge that requires urgent attention and the increasing trend in its cases is suggested to continue for more decades. Currently, there is no permanent cure for DM. Many treatment regimens have shown promising results in DM management. Yet, notwithstanding the potential of these giant treatment plans, DM remains a serious challenge that may continue to threaten public health. Thus, the problems encountered in each of these approaches need to be addressed to achieve a robust, efficient, and safe clinical management plan. There is a need for optimal metabolic regulation of glucose, blood pressure, and body weight which requires proper education and support for the improvement of diet, physical activity, and reduction in body weight. To effectively and successfully manage the control of this disease, an emphasis on public policies to reinforce health care access and resources, the promotion of a patient-centred care approach, and health-promoting infrastructures at environmental level are required.

## Figures and Tables

**Table 1:** Promising targets that can be employed for T2DM gene therapy.

Class	Genes	Main Function
Genes modulating homeostasis of glucose	GLUTs	Involved in the re-absorption of filtered glucose from the kidney into the bloodstream
	SGLTs	Partake profoundly in muscle and hepatic glucose fluxes
	FGFs	Functions significantly in the homeostasis of glucose
	SIRT6	Connected with an expression of GLUTs and increased glycolysis
Genes enhancing the secretion of insulin and/or sensitivity	GLP-1 and its analogs/agonists	Boost the survival of beta-cell, provoke the expression of the insulin gene, and synthesis
	GPGRs and their agonists	Enhances the secretion of insulin and GLP-1
	CTB-APSL	Enhances secretion of insulin and insulin resistance
	IKK E, TBK1	Linked with diminution in weight, insulin resistance, fatty liver as well as inflammation
Genes attenuating diabetic induced complications	IL-1b	Linked with inflammation and b-cell failure
	ADPN	Attenuates diabetic nephropathy
	TGF-a	Has a function in DKD linked with nephron reduction
	NLRP3	Attenuates diabetic cardiomyopathy
	CDKN2A/2B	connected with modulation of T-cell phenotype and chronic inflammation
	HSP70	Connected with bioenergetics of mitochondrion and diabetic sensory neuropathy
	MicroRNAs	Implicated in the modulation of diabetic microvasculature

Legend: HSP70 = heat shock protein 70; NLRP3 = nucleotide-binding oligomerization domain-like receptor protein 3; SGLTs = sodium-glucose co-transporters; GLUTs, glucose transporters; SIRT6=Sirtuin 6; FGFs = fibroblast growth factors; GPGRs = G protein–coupled receptors; GLP-1= glycogen-like peptide 1; ADPN = adiponectin; CTB APSL = cholera toxin B subunit and active peptide from shark liver; TGF-a = transforming growth factor-alpha; DKD = diabetic kidney disease [51].

## Data Availability

Not applicable.
